# The functional role of cellular senescence during vascular calcification in chronic kidney disease

**DOI:** 10.3389/fendo.2024.1330942

**Published:** 2024-01-22

**Authors:** Ya-Ping Fang, Yu Zhao, Jia-Yi Huang, Xin Yang, Yan Liu, Xiao-Liang Zhang

**Affiliations:** ^1^ Institute of Nephrology, Zhong Da Hospital, Southeast University School of Medicine, Nanjing, Jiangsu, China; ^2^ Department of Clinical Medicine, Southeast University School of Medicine, Nanjing, Jiangsu, China

**Keywords:** vascular calcification, senescence, chronic kidney disease, vascular smooth muscle cells, vascular endothelial cells, vascular progenitor/stem cells, macrophages

## Abstract

Vascular calcification (VC) has emerged as a key predictor of cardiovascular events in patients with chronic kidney disease (CKD). In recent years, an expanding body of research has put forth the concept of accelerated vascular aging among CKD patients, highlighting the significance of vascular cells senescence in the process of VC. Within the milieu of uremia, senescent vascular endothelial cells (VECs) release extracellular microvesicles (MV) that promote vascular smooth muscle cells (VSMCs) senescence, thereby triggering the subsequent osteogenic phenotypic switch and ultimately contributing to the VC process. In addition, senescent vascular progenitor or stem cells with diminished ability to differentiate into VECs and VSMCS, compromise the repair of vascular integrity, on the other hand, release a cascade of molecules associated with senescence, collectively known as the senescence-associated secretory phenotype (SASP), perpetuating the senescence phenomenon. Furthermore, SASP triggers the recruitment of monocytes and macrophages, as well as adjacent VECs and VSMCs into a pro-adhesive and pro-inflammatory senescent state. This pro-inflammatory microenvironment niche not only impacts the functionality of immune cells but also influences the differentiation of myeloid immune cells, thereby amplifying the reduced ability to effectively clear senescent cells of senescent macrophages, promoted calcification of VSMCs. The objective of this paper is to provide a comprehensive review of the contribution of vascular cell senescence to the emergence and advancement of VC. Gaining a comprehensive understanding of the involvement of cellular senescence within the vessel wall is pivotal, especially when it comes to its intersection with VC. This knowledge is essential for advancing groundbreaking anti-aging therapies, aiming to effectively mitigate cardiovascular diseases.

## Graphical Abstract

Overview of the role of cellular senescence in regulating vascular calcification. The complex landscape of CKD hastens the senescence of vascular endothelial cells (VECs) and undermines their functional integrity, exhibiting a distinctive profile characterized by the reduced expression of nitric oxide (NO) and an elevation in reactive oxygen species (ROS). Senescent VECs release microvesicles (MVs) to trigger senescence and calcification in VSMCs. On the other hand, senescent VECs attract monocytes to the endothelium and induce the proliferation and migration of VSMCs. Pro-inflammatory phenotypic macrophages subsequently promote VSMC calcification by stimulating carbonic anhydrase I (CA1) and CA2 via secreting TNFα, or NLRP3 inflammasome. Mesenchymal stromal cells (MSCs) and adventitial fibroblasts (AFs) exhibit a potential for differentiation, with the ability to transform into VECs and VSMCs, contributing to the replenishment of senescent cells. However, the proliferation and differentiation capacities of senescent MSCs and AFs are diminished.

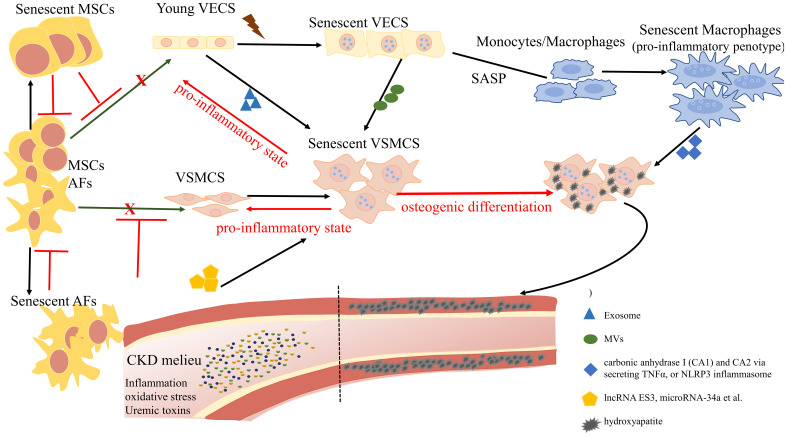

## 1 Introduction

Cardiovascular disease is the leading cause of death in patients with chronic kidney disease (CKD), a reality that persists even during the initial CKD stages (CKD stages 1-3). Vascular calcification (VC) emerges as both an independent predictor and a pivotal driving force behind the onset and progression of cardiovascular disease in this patient demographic (1). Despite significant advancements in VC drug development, such as SNF472 and sodium thiosulfate, the incidence of cardiovascular mortality among CKD patients persists at elevated levels.

There is a growing focus on the role of senescent cells, particularly in the context of accelerated vascular aging among CKD patients, with VC emerging as a significant phenotype of this aging process (2). Accumulating data suggests that the transition of pro-calcificatory/osteoblastic phenotype of senescent vascular smooth muscle cells (VSMCs) play a vitol role. However, the deviation between vascular aging and chronological aging still need further invesatigated.

Improving our understanding of the interplay between aging and VC is of utmost importance. To gain a comprehensive understanding of the pathophysiological processes driving VC and to develop prospective therapies for CKD patients with VC, this review explores the current knowledge of VC in CKD, with a particular emphasis on the role of vascular cell senescence and cellular interactions in the progression of VC.

## 2 Vascular calcification

The type of VC that develops depends on its location, and it commonly takes the following forms: intimal calcification, media calcification, and valve calcification. Intimal calcification often presents as atherosclerosis, primarily impacting larger and medium-sized arteries along with their branches. This condition arises from chronic vascular inflammation and involves the infiltration of vascular smooth muscle cells (VSMCs) and macrophages in the lipid-rich regions of atherosclerotic plaques. Ischemic heart disease stems from the formation, rupture, and obstruction of these plaques within arteries (3). Monckeberg’s sclerosis, also known as medial arterial calcification (MAC), is an age-related degenerative aiflment first proposed by Johann Georg Monckeb in 1903 (4). The hallmark of MAC is the diffuse and continuous deposition of hydroxyapatite in the medial layer (5). The presence of MAC can be observed in the walls of vessels with varying internal diameters, which may experience stiffness. This stiffness often leads to increased pulse wave velocity (PWV), reduced blood flow to the heart (cardiac hypoperfusion), elevated systolic blood pressure, left ventricular hypertrophy, and eventually heart failure (4–6). MAC is frequently associated with abnormalities in calcium and phosphorus metabolism, as well as mineral and bone metabolism, the severity of CKD, dialysis vintage, aging (7), diabetes (5), and rapid progression of medial calcification in patients with end-stage renal disease who are undergoing hemodialysis (8).

In contemporary medical practices, conventional X-rays, ultrasonography, and computed tomography (CT) scans have been used for detecting and monitoring calcification. Additionally, circulating biomarkers, which reflect pathophysiological changes, thus contribute valuable insights. However, despite these advancements, the clinical challenge in the context of CKD persists due to the limited sensitivity of VC diagnosis (9). Previously, it was believed that VC occurred passively, involving the deposition of calcium and phosphate as hydroxyapatite in the arterial wall. However, mounting evidence now suggests that VC is an active, highly regulated, and resembling the bone-forming process known as osteogenesis. This active process is driven by a osteogenic-like phenotypic transformation of VSMCs (5). Beyond, a range of factors contribute to the complex process of VSMCs calcification, including imbalances in the regulation of calcium and phosphate levels, cell death (apoptosis, ferroptosis), diminished presence of calcification inhibitors, the release of vesicles containing mineral-forming components (mineralizing matrix vesicles or MVs), and crosstalk between VSMCs and VECs (10–12). In an environment with high calcium levels, the quantity of calcium-sensing receptors (CaSRs) on the surface of VSMCs decreases, resulting in an increase in intracellular calcium content. This heightened calcium concentration triggers apoptosis in VSMCs, leading to the formation of hydroxyapatite deposits sites from these apoptotic cells. The release of calcium from dying cells perpetuates apoptosis, thus accelerating the vicious cycle of VC (13, 14). Elevated levels of phosphorus have a direct effect, triggering monocytes to synthesize and release tumor necrosis factor-alpha (TNF-α) via the PiT-1 pathway. This initiates an inflammatory state, furthering the transformation of VSMCs into an osteogenic phenotype and the subsequent calcification process (15). As a result, VSMCs shift toward a cell phenotype resembling that of osteoblasts, leading to an increase in the expression of regulatory proteins like Runx2, Osterix, Msx2, and Sox9. Additionally, these cells release mineralized MVs while reducing the presence of endogenous calcification inhibitors within these vesicles, such as MGP (16–18). Moreover, VSMCs produce alkaline phosphatase (ALP), which deactivates the mineralization-inhibiting effects of pyrophosphate, releasing free phosphate that serves as a building block for VC (19).

Recent advancements highlight abnormalities in epigenetic modifications (such as DNA methylation, histone modifications, and noncoding RNAs), ferroptosis of VSMCs, autophagy dysfunction, and senescence as active participants in the cellular processes leading to VC (12, 20, 21). Extensive studies have delved into the involvement of senescent vascular cells, including VECs, VSMCs, and macrophages, in propagating senescence through paracrine mechanisms (e.g., SASP and MV) (22–24). Through immunostaining and analysis of protein expression in abdominal aortic samples from 61 kidney transplant recipients, it was disclosed that CKD patients exhibiting coronary artery calcification, showed notably elevated levels of p16. Remarkably, the count of cells displaying both p16 and SA-β-Gal-positivity exhibited a positive correlation with the severity of medial calcification in CKD (25). Furthermore, eliminating senescent cells yielded significant reductions in aortic aging markers and osteogenic marker, osterix (26). The burden of senescent cells may drive the VC process.

## 3 Cellular senescence

Cellular senescence forms the cornerstone of organismal aging. Professor Leonard Hayflick’s breakthrough discovery in the 1960s revealed that human diploid cells, when cultured *in vitro*, possess a finite capacity for proliferation, eventually culminating in an irreversible state of growth arrest irrespective of culturing conditions. This phenomenon, termed cellular senescence, defines the maximum number of cellular divisions, referred to as the Hayflick limit (27, 28). Cellular senescence entails the irreversible and enduring cessation of cell division, accompanied by observable morphological alterations such as cell flattening, expanded cytoplasm, and nucleus changes. Cellular senescence is currently divided into two main categories: replicative senescence (RS) and stress-induced premature senescence (SIPS). Replicative senescence occurs during normal physiological processes when cells continuously replicate, leading to telomere shortening and cell exit from the cell cycle, which also represents a cause for the Hayflick limit. However, replication is not the only factor causing telomere shortening, environmental stress can indeed lead to telomere shortening (29). SIPS refers to aging that occurs in cells before the Hayflick limit in response to various environmental stimuli. Stimulating factors include excessive activation of oncogenes, loss of tumor suppressor factors, damage to DNA or chromosome structures, mitochondrial dysfunction, oxidative stress, wound healing, various cytokines, and intercellular signal transduction (30). This process is also marked by the expression of the senescence-associated secretory phenotype (SASP), which bridges cellular senescence with tissue dysfunction and organismal aging (27, 31). The SASP components exhibit strong autocrine activity and can also transmit senescence via paracrine signaling to adjacent cells. This characterization creates an pro-inflammatory microenvironment, resulting in a persistent low-level chronic inflammation, a state known as inflammaging, and causing age-related diseases (32). Indeed, senescent cells play a dual role: they not only generate a pro-inflammatory microenvironment primarily through the senescence-associated SASP but also impede stem cell proliferation and regeneration. This dual action serves to blunt the tissue regeneration and repair processes, further complicating the overall health and recovery mechanisms (33). On one hand, senescent cells display anti-proliferative attributes that prevent the propagation of damaged cells, thus averting the development of potential malignant cells. Additionally, senescent cells release SASP factors that extend senescence to neighboring healthy cells while recruiting immune cells for removal (34). On the other hand, senescent cells express BCL-2 family proteins that confer resistance to apoptosis (35), allowing them to amass within the body as age advances, and ultimately leading to the progression of age-related diseases.

**Figure 1:**
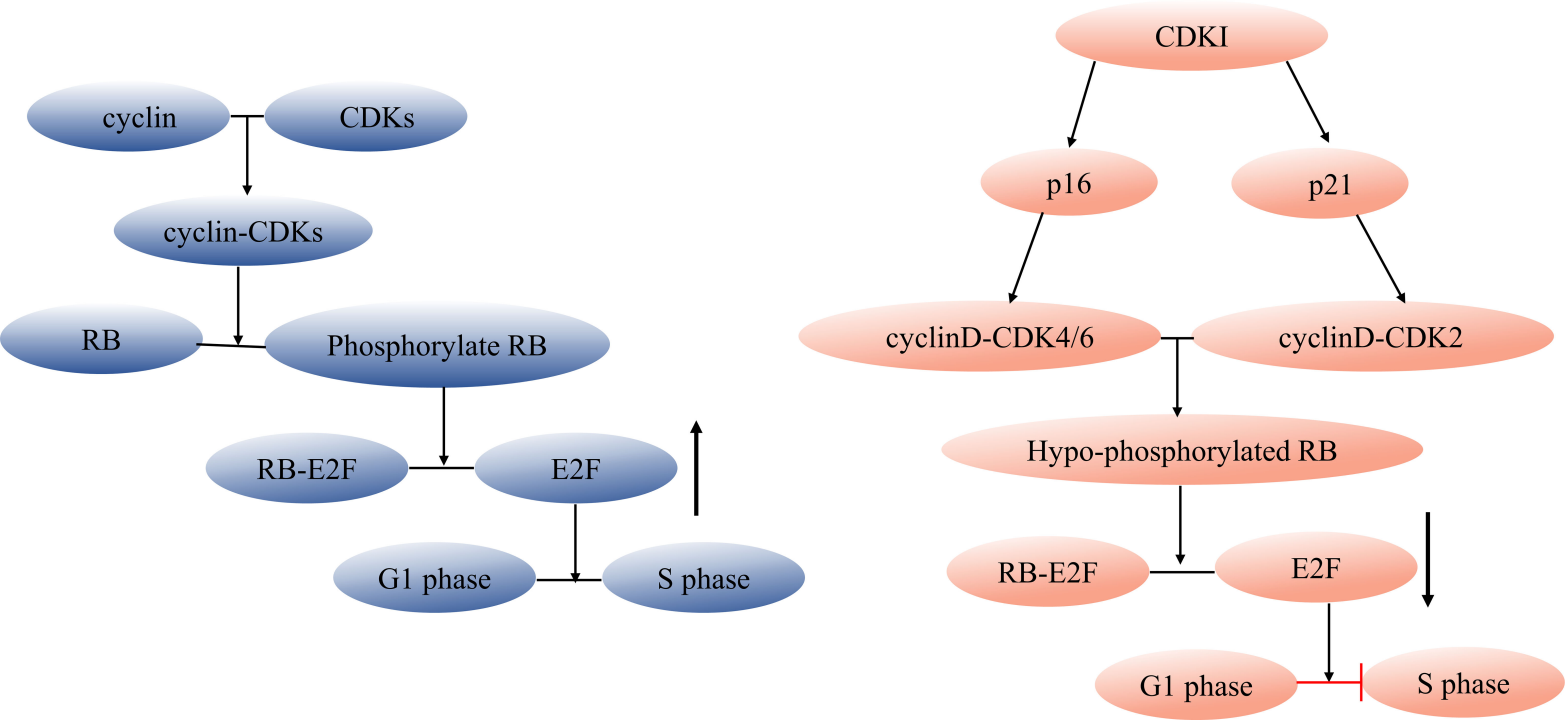
Regulation of cell cycle and senescence pathways. During cell division, cyclin and CDK combine forming a cyclin-CDK complex that initiates the downstream phosphorylation of RB. The phosphorylated RB then disengages the transcription factor E2F from the RB-E2F complex, facilitating DNA transcription and propelling the cell cycle into the S phase. CDKI can inhibit this process. Cell cycle arrest during cellular senescence primarily hinges on the p53/p21 and p16/RB pathways (47, 48), p53 acts as an upstream regulator of p21, while both p16 and p21 belong to the CDKI family. These members inhibit cyclinD-CDK4/6 and cyclinE/A-CDK2, respectively, tightly tethering low-phosphorylated RB to E2F and halting cells in the G1 phase. CDK, cyclin-dependent kinase, CDKI, cyclin-dependent kinase inhibitor.

Targeting the elimination of senescent cells as a strategy to alleviate age-related diseases has been a focal point for scientists. The use of senolytics, a cocktail designed to selectively clear senescent cells, has shown promising outcomes. By reducing the number of senescent cells, this approach not only ameliorates physical dysfunction but also decreases mortality hazard and enhances healthspan, providing direct evidence that cellular senescence is a causative factor in age-related diseases (36). In the field of Geroscience, researchers advocate for a shift in perspective by focusing on the aging process itself rather than individual diseases. This approach holds the potential to delay or even halt the development of age-related diseases (37). However, the complexity of cellular senescence poses challenges, particularly in identifying specific biomarkers for senescent cell detection. Researchers have made strides in this regard, developing tools such as DNAm PhenoAge, inflammatory aging clock (iAge), and others, to predict physiological age. Since the introduction of the epigenetic clock by Prof. Horvath (38–40), various methods combining multiple markers have been employed to confirm the occurrence of cellular senescence at the cellular level. These markers encompass senescence-associated β-galactosidase (SA-β-gal), cell cycle-dependent protein kinase inhibitors (p16, p21, p53), cell proliferation indicators (Ki67), senescence-associated heterochromatin foci (SAHF), DNA damage response, and SASP (41–43). The cell cycle arrest that characterizes cellular senescence is primarily governed by the p53/p21 and p16/RB pathways (44, 45). Notably, p53 functions upstream of p21, while p16 and p21 belong to the cyclin-dependent kinase inhibitor (CDKI) family, inhibiting cyclinD-CDK4/6 and cyclinE/A-CDK2, respectively. This action binds minimally phosphorylated RB closely to E2F, halting cells in the G1 phase (46) (**Figure 1**). It is important to acknowledge that p21 is mainly expressed in the early stages of senescence induction and does not persist, while p16 is not highly expressed in all senescent cells (49–51). While SASP is a prevalent feature of senescent cells, its composition is not entirely fixed. Components of SASP differ due to variations in cell characteristics, stimulus methods, and senescence duration (52). Nevertheless, the significance of employing a combination of multiple markers to identify cellular senescence cannot be emphasized enough.

## 4 Role of cell senescence in vascular calcification: insights from chronic kidney disease

In 1989, Professor Virchow first introduced a captivating discovery: individuals suffering from CKD manifest the same pathological transformations of medial calcification commonly observed in the elderly population (53). Foley et al. made a comparable observation regarding vascular traits among young hemodialysis patients, likening them to those found in individuals in their eighties and nineties (54). A study featured in NEJM in 2000 disclosed that the occurrence of VC among young hemodialysis patients aged 20 to 30 years reached a staggering 87.5% (14 out of 16), markedly surpassing the corresponding age group in the general population (3.3%, 2 out of 60) (55). Furthermore, Girndt et al. proposed that the extent of VC could serve as an indicator of an individual’s biological age (2). Collectively, these findings indicate an accelerated vascular aging progress in CKD patients.

In the realm of CKD, a milieu defined by the presence of uremic toxins, inflammatory processes, and oxidative stress persists, impervious even to renal transplantation. This environment fosters a state of premature aging, a condition further exacerbated by hemodialysis, which accelerates telomere attrition (56, 57). Within this complex scenario, the burden of senescent cells emerges as a substantial factor contributing to VC in the CKD milieu. Notably, interventions aimed at eliminating senescent cells using genetic approaches or dasatinib plus quercetin (D+Q) yielded significant reductions in aortic aging markers and calcification in aging and atherosclerotic mice. This was accompanied by a downregulation of the osteogenic marker, osterix (26). Furthermore, the pro-inflammatory secretome (SASP) derived from senescent cells has been found to elevate in both uremic mouse models and patients (58, 59). However, the direct impact of SASP on vascular cells remains underexplored, with potential mechanisms possibly involving the propagation of senescence or amplification of localized inflammation. However, numerous studies have shown that sirtuins, histone deacetylases, or known as the family of longevity proteins, play a protective role in VC. For example, sirtuin 6 mediated the deacetylation of Runx2, which subsequently facilitated its nuclear export via exportin 1 (XPO1). This event eventually triggered the degradation of Runx2 through the ubiquitin-proteasome system, thereby inhibiting the osteogenic transdifferentiation of VSMCs (60). Additionally, sirtuin 1 was upregulated by spermidine, thus alleviating VC in CKD (61). Moreover, compelling evidence suggests a decline in nicotinamide adenine dinucleotide (oxidized form, NAD+) levels in both CKD and acute kidney injury (AKI). This molecule plays a pivotal role as a coenzyme in crucial cellular metabolic activities. The supplementation of its precursor, nicotinamide mononucleotide (NMN), has demonstrated positive effects on physical endurance and the maintenance of biological age (62). The popularity of NMN is significant due to its potential to activate longevity proteins, improve mitochondrial metabolism, and contribute to the repair of damaged DNA. In the near future, there is hope that NMN could become a promising choice for extending healthy lifespan. Furthermore, nuclear factor erythroid 2-related factor 2 (NRF2) has emerged as a proposed protective factor against calcification and aging. Its transcriptional suppression of pro-inflammatory genes and antioxidant effects contribute to its role in mitigating these processes (63).

Senescent cells are not quiescent, nonfunctional and may be continuously releasing inflammatory mediators, microparticles, etc., affecting the level of transcription, post-transcriptional regulation, translation, and metabolism by autocrine/paracrine means, which in turn affects phenotypic transformation and microenvironmental niche on itself or adjacent cells, resulting acceleration of calcification. The intricate association between aging and VC implies that delving deeper into their interaction could potentially pave the way for novel avenues in diagnosing and treating VC.

## 5 Role of vascular cell senescence in vascular calcification

Both VECs and VSMCs constitute essential cellular components residing within the intimal and medial layers of blood vessels, respectively. The adventitial layer, on the other hand, is comprised of a diverse array of cells, fibroblasts, lymphocytes, vascular progenitors/stem cells, pericytes, and primarily monocytes/macrophages-based immune cells. This rich composition could potentially open novel avenues for advancing the diagnosis and treatment of VC. A growing body of evidence is highlighting the pivotal role played by adventitial cells in blood vessel remodeling and the maintenance of vascular homeostasis (64, 65). Interestingly, adventitial fibroblasts (AFs) exhibit the remarkable ability to transdifferentiate into VSMCs in response to microenvironmental cytokines, subsequently migrating to the luminal side of the vessel (66). Recent advancements have shed light on the role of exosomes derived from AFs, predominant cells in the adventitia. These exosomes expedite VSMC calcification by delivering miR-21-5p, which, in turn, inhibits cysteine-rich motor neuron 1 (Crim1) (67). Conversely, adventitial pericytes (APC) counteract calcification by triggering an anti-calcific effect through the upregulation of microRNA-132-3p (68). Cells constitute the fundamental constituents of tissues, organs, and organisms. And it is the progression of cellular senescence that propels the aging process across these levels.

### 5.1 Role of vascular endothelial cell senescence in vascular calcification

VECs adeptly sense a myriad of stimuli coursing through the bloodstream and promptly respond, upholding vascular equilibrium via autocrine or paracrine mechanisms. These mechanisms play a role in regulating diverse functions, including blood pressure maintenance, promotion of angiogenesis, and coagulation regulation (69). In the complex landscape of CKD, characterized by heightened inflammation, oxidative stress, leukocyte migration, adhesion, cell death, and the emergence of a thrombotic phenotype, an array of circulating molecules holds sway over endothelial equilibrium These molecules exert their influence through frequently activated cellular signaling pathways, including ROS, MAPK/NF-κB, Aryl-Hydrocarbon Receptor, and RAGE pathways (70). A noteworthy phenomenon within this milieu is the endothelial-to-mesenchymal transition (EndMT) of VECs. This transition involves endothelial cells adopting a mesenchymal-like state, providing a conceptual link to the generation of osteogenic cells that contribute to VC. Wang et al. proposed that EndMT induced by Parathyroid hormone (PTH) plays a role in VC through the miR-29a-5p/GSAP/Notch1 pathway in CKD rats (71). Notably, indoxyl sulfate (IS) and sulfate paracresol emerge as significant players, hastening the senescence of endothelial cells and undermining their functional integrity (72). Senescent VECs exhibit a distinctive profile characterized by reduced expression of nitric oxide (NO) and an elevation in reactive oxygen species (ROS) (73). Moreover, the accumulation of senescent VECs suppresses the expression of adherens junction proteins and compromises the endothelial migration potential in non-senescent VECs (74). This intricate interplay underscores the impact of senescence on the functional and molecular dynamics within the endothelial environment. Given the spatial proximity of endothelial cells and VSMCs, an intriguing query arises: could endothelial cells, especially senescent ones, exert an impact on VSMC calcification? Intriguing *in vitro* findings add credence to this question. Under high glucose stimulation, endothelial cells have been observed to release exosomes housing Notch3 or versican proteins. This, in turn, triggers senescence and calcification in VSMCs (10, 11). Further studies reveal that senescent endothelial cells possess the ability to attract monocytes to the endothelium and induce the proliferation and migration of VSMCs (47). Additionally, the release of microvesicles (MVs) from senescent endothelial cells emerges as a key factor driving VSMC senescence and calcification. Noteworthy is the intervention using IS on human umbilical vein endothelial cells (HUVECs), which results in EC senescence and subsequent MV release. These MVs are identified as the primary instigators behind human aortic smooth muscle cells (HASMCs) calcification, coupled with the upregulation of pro-inflammatory factors (22). Furthermore, the influence of senescent endothelial cells and their derived MVs does not end there. Examination of plasma from elderly individuals and MVs sourced from senescent HUVECs underscores their promoting effect on HASMC calcification. These MVs are characterized by the upregulated expression of membrane-associated proteins A2, A6, and BMP2 (48).

### 5.2 Role of vascular smooth muscle cell senescence in vascular calcification

The transformation of VSMCs into osteogenic phenotypes stands as a pivotal cellular mechanism underlying VC (75). When VSMCs undergo senescence, they adopt a pro-calcification and osteogenic phenotype (76, 77). Microarray analysis of senescent VSMCs cultured *in vitro* unveiled distinct gene expression patterns related to VC including bone morphogenetic protein-2 (BMP-2), osteoprotegerin (OPG), osteopontin (OPN), secreted phosphoprotein 1 (SPP1), and matrix Gla protein (MGP) (76). Senescent VSMCs exhibit upregulation of RUNX2, while knockdown of RUNX2 resulted in a significant reduction of osteoblast markers, as well as in calcification (77). Furthermore, senescent VSMCs exhibit the expression of MMP9 and SASP, which in turn triggers adjacent ECs and VSMCs to assume a pro-adhesive and pro-inflammatory state. This cascade contributes to the establishment of chronic vascular inflammation (78).

Within the context of CKD, VSMCs encountered by patients face an array of pro-calcification environments. These include reactive oxygen species (ROS), elevated calcium and phosphate levels, and the influence of uremic toxins. This multifaceted assault can induce DNA damage in VSMCs. If left unresolved, persistent DNA damage prompts VSMCs to enter a state of senescence. The release of SASP, including BMP2, IL-6, and OPG, by senescent cells further spurs VSMCs toward osteogenic differentiation (23). Notably, the uremic toxin IS triggers upregulation of p21, p53, and nuclear lamin A in VSMCs through pathways driven by oxidative stress (79). The landscape of molecular mechanisms associated with VSMC senescence-induced calcification is intricate and multifaceted. For instance, the downregulation of microRNA-34a culminates in the inhibition of Sirtuin1 and the AXL receptor tyrosine kinase, thereby promoting both VSMC senescence and calcification (80, 81). Another intriguing facet is the involvement of long non-coding RNA (lncRNA) ES3, which binds to Bhlhe40. This interaction effectively silences multiple small interfering RNAs and subsequently ushers in the era of VSMC senescence and calcification (82).

### 5.3 Role of vascular progenitor/stem cell depletion in vascular calcification

The adventitia hosts an array of vascular progenitor/stem cells, encompassing mesenchymal stromal cells (MSCs), AFs, and pericytes. These vascular progenitor/stem cells exhibit a versatile potential for differentiation, capable of transforming into VECs and VSMCs. They play a pivotal role in vascular repair and remodeling following vascular injury and in the replenishment of senescent cells. The emergence of MSCs has ignited optimism within regenerative medicine, due to their remarkable attributes such as ease of procurement, isolation, immune regulation, and multi-lineage differentiation capabilities (83). Notably, MSC-derived extracellular vesicles (EVs) have demonstrated the ability to alleviate VEC senescence through microRNA-146a, thereby diminishing SASP expression while stimulating angiogenesis (84). Stem cell senescence is also a prominent theory of organismal aging (10). The depletion of vascular progenitor/stem cells can engender a decline in the self-renewal potential of VECs and VSMCs, potentially setting the stage for vascular aging and the jsubsequent advancement of age-associated vascular disorders (85).

Similar to senescent cells, senescent MSCs undergo distinct morphological alterations characterized by flattening and enlargement. Concomitantly, their proliferation and differentiation capacities wane (83). Senescent MSCs also express pro-inflammatory SASP, which inhibits the proliferation of hematopoietic stem cells, thus expediting their premature senescence (86). This, in turn, indirectly participates in the aging and calcification trajectory of blood vessels. Senescent AFs, akin to their cellular counterparts, release SASP laden with pro-inflammatory and pro-tissue degradation factors, thus perpetuating the senescence phenomenon (87). Aged AFs exhibit significant differential expression of pro-inflammatory and osteogenic genes via transcriptional analysis (88). Moreover, in patients with calcification associated with rheumatoid arthritis, the circulating osteogenic endothelial progenitor cells (EPC) are related to vascular aging, encountering a decline in both number and differentiation potential, culminating in hastened cellular senescence (89, 90). A distinct viewpoint is proposed by Wu et al., indicating that the proliferative and anti-apoptotic capabilities of aged adipose stem cells tend to outshine those of bone marrow stem cells (91). Consequently, adipose-derived stem cells may offer greater suitability for autsue and organismal aging, and chronic age-related conditions. Recent research underscores the potential of inhibiting the immune checkpoint protein programmed death-ligand 1 (PD-L1) in senescent cells. Such intervention appears to ameliorate the inflammatory state and mitigate age-related manifestations (92). Zhang et al. found that senescence characteristics of hematopoietic stem cells can be reversed and their long-term multilineage reconstitution capacity restored by harnessing matrix stiffness (93). In addition, senescent cells do not invariably induce detrimental effects, indeed, p16^INK4a+^ fibroblasts can actually foster the regeneration of epithelial stem cells (94). Although research on the role of vascular progenitor/stem cell senescence in VC remains limited, delving deeper into this avenue could yield strategies for anti-aging therapies aimed at postponing calcification processes. This unexplored territory holds promising potential for shedding light on the intricate intersections of senescence, vascular health, and aging-related outcomes.

### 5.4 Role of macrophage senescence in vascular calcification

Immunosenescence, the progressive decline in immune function with age, represents a causal contributor to systemic aging (95). This phenomenon is marked by a shift in immune cell differentiation, favoring myeloid lineage development while diminishing lymphoid lineage differentiation (96). As a result, immune cells experience a reduced ability to effectively clear senescent cells from the body, leading to the accumulation of these aged cells. Consequently, the role of immune cell senescence in age-related diseases has garnered substantial attention.

As mentioned above, specific components of the SASP are adept at recruiting immune cells. Significant contributors such as IL-6, IL-7, IL-8, IL-1α, IL-1β, TNF-α, macrophage colony-stimulating factor (M-CSF), G-CSF, and granulocyte-macrophage colony-stimulating factor (GM-CSF) exert their influence, drawing in a range of immune players including macrophages, granulocytes, neutrophils, and T lymphocytes (97). Immunosenescence results may lead to myeloid lineage bias, prompting us to consider that macrophages might indeed hold a central position in the realm of aging-related diseases. This notion finds its roots in the theory of inflamm-aging, which initially highlighted the pivotal role of macroph-aging – the gradual activation of macrophages as time advances (98). Macrophages, critical constituents of innate immunity, exhibit a tendency to shift from an anti-inflammatory M2 phenotype to a pro-inflammatory M1 phenotype with advancing age (99), and subsequently promote VSMCs calcification by sitimulating carbonic anhydrase I (CA1) and CA2 via secreting TNFα, or NLRP3 inflammasome (100, 101). Calcified smooth muscle cells induce the differentiation of macrophages into osteoblasts through RANKL, fostering a positive feedback loop that promotes VC (102). In a dystrophic muscle pathology model, the accumulation of pro-inflammatory M1 macrophages at ectopic calcification sites was evident. This accumulation was accompanied by the upregulation of senescence markers such as p21, C12FDG, and SASP (24). However, the specific molecular mechanisms by which senescent macrophages induce VC remain unclear, and require further investigation.

## 6 Summary and conclusions

Throughout the extensive trajectory of human evolution, cellular senescence emerges as the ultimate choice of cells when confronted with diverse physical and chemical stimuli, carefully weighing the pros and cons. While it acts as a brake on the proliferation of aberrant cells, it concurrently propels the advancement of age-related diseases. Notably, cardiovascular incidents and mortality rates maintain a distressingly high presence in patients with CKD. Within this context, the process of vascular premature senescence stands as a pivotal player in the intricate symphony of calcification. This article mainly focused on the multifaceted role of senescence across various vascular cell types, encompassing endothelial cells, vascular smooth muscle cells, vascular progenitor/stem cells, and macrophages. The ultimate aim is to distill the current corpus of literature, culminating in an informative synthesis that paves the way for prospective endeavors in the realm of anti-aging therapies, specifically designed to counteract and alleviate the menace of VC.

Senolytics, such as D+Q, fisetin (103), and procyanidin C1 (PCC1) (104), among others, have experienced continuous expansion and have even entered clinical trials since Professor Kirkland and colleagues discovered the anti-aging effects of D+Q in 2015 (105). Currently, D+Q appears to be the most promising agent (106, 107). However, it is noteworthy that many senolytics operate by suppressing antiapoptotic signal pathways in senescent cells, potentially increasing the risk of off-target effects on healthy tissues. To address this concern, chimeric antigen receptor (CAR) T cells have shown promise in selectively and effectively removing senescent cells by identifying proteins broadly induced in these cells (108, 109). Additionally, Senolytic vaccination, targeting glycoprotein nonmetastatic melanoma protein B (GPNMB), has successfully extended lifespan) in progeroid mice (about 20%) (110). The challenge lies in the variations in aging characteristics among species, individuals, organs, and even cells. Tailoring anti-aging regimens specifically to senescent cells is a future direction of exploration in this field. Despite these challenges, the development of a gene set (SenMayo) has made it possible to identify senescent cells *in vivo*. Moreover, the application of deep learning to discover senotherapeutics holds promise for the future (111, 112).

## Author contributions

Y-PF: Data curation, Investigation, Visualization, Writing – original draft. YZ: Funding acquisition, Supervision, Writing – review & editing. J-YH: Data curation, Writing – review & editing. XY: Data curation, Writing – review & editing. YL: Data curation, Writing – review & editing. X-LZ: Funding acquisition, Supervision, Writing – review & editing.
